# Exercise effects on bed rest-induced brain changes

**DOI:** 10.1371/journal.pone.0205515

**Published:** 2018-10-11

**Authors:** Vincent Koppelmans, Jessica M. Scott, Meghan E. Downs, Kaitlin E. Cassady, Peng Yuan, Ofer Pasternak, Scott J. Wood, Yiri E. De Dios, Nichole E. Gadd, Igor Kofman, Roy Riascos, Patricia A. Reuter-Lorenz, Jacob J. Bloomberg, Ajitkumar P. Mulavara, Lori L. Ploutz-Snyder, Rachael D. Seidler

**Affiliations:** 1 School of Kinesiology, University of Michigan, Ann Arbor, Michigan, United States of America; 2 Department of Psychiatry, University of Utah, Salt Lake City, Utah, United States of America; 3 Memorial Sloan Kettering Cancer Center, New York, New York, United States of America; 4 Universities Space Research Association, NASA Johnson Space Center, Houston, Texas, United States of America; 5 KBRwyle, Houston, Texas, United States of America; 6 Department of Psychology, University of Michigan, Ann Arbor, Michigan, United States of America; 7 Department of Psychiatry and Radiology, Brigham and Women’s Hospital, Harvard Medical School, Boston, Massachusetts, United States of America; 8 NASA Johnson Space Center, Houston, Texas, United States of America; 9 The University of Texas Health Science Center, Houston, Texas, United States of America; 10 Neuroscience Program, University of Michigan, Ann Arbor, Michigan, United States of America; 11 Department of Applied Physiology & Kinesiology, University of Florida, Gainesville, Florida, United States of America; McLean Hospital, UNITED STATES

## Abstract

**Purpose:**

Spaceflight negatively affects sensorimotor behavior; exercise mitigates some of these effects. Head down tilt bed rest (HDBR) induces body unloading and fluid shifts, and is often used to investigate spaceflight effects. Here, we examined whether exercise mitigates effects of 70 days HDBR on the brain and if fitness and brain changes with HDBR are related.

**Methods:**

HDBR subjects were randomized to no-exercise (n = 5) or traditional aerobic and resistance exercise (n = 5). Additionally, a flywheel exercise group was included (n = 8). Exercise protocols for exercise groups were similar in intensity, therefore these groups were pooled in statistical analyses. Pre and post-HDBR MRI (structure and structural/functional connectivity) and physical fitness measures (lower body strength, muscle cross sectional area, VO_2_ max, body composition) were collected. Voxel-wise permutation analyses were used to test group differences in brain changes, and their associations with fitness changes.

**Results:**

Comparisons of exercisers to controls revealed that exercise led to smaller fitness deterioration with HDBR but did not affect brain volume or connectivity. Group comparisons showed that exercise modulated post-HDBR recovery of brain connectivity in somatosensory regions. Posthoc analysis showed that this was related to functional connectivity decrease with HDBR in non-exercisers but not in exercisers. Correlational analyses between fitness and brain changes showed that fitness decreases were associated with functional connectivity and volumetric increases (all *r* >.74), potentially reflecting compensation. Modest brain changes or even decreases in connectivity and volume were observed in subjects who maintained or showed small fitness gains. These results did not survive Bonferroni correction, but can be considered meaningful because of the large effect sizes.

**Conclusion:**

Exercise performed during HDBR mitigates declines in fitness and strength. Associations between fitness and brain connectivity and volume changes, although unadjusted for multiple comparisons in this small sample, suggest that supine exercise reduces compensatory HDBR-induced brain changes.

## Introduction

During spaceflight astronauts adapt to microgravity. Upon return to Earth they often experience problems with posture control [1] and locomotion [2]. These motor behavioral effects are linked to altered leg muscle activation patterns, head-trunk coordination [3], and adaptive central reinterpretation of visual, vestibular and proprioceptive information [4–6]. To date, four neuroimaging studies in astronauts have been published. These studies which all had longitudinal designs demonstrated that spaceflight can lead to structural [7–9] and functional brain changes, including in regions that are involved in motor control [10]. The structural brain changes that we reported previously were observed throughout the brain and were mostly gray matter volume decreases and could to a certain extent reflect cerebral fluid shifts [11]. However, specific gray matter volume increases were observed in specific regions important for movement of the lower limbs [8].

Head down tilt bed rest (HDBR) is widely used as a spaceflight analog research environment on Earth. It mimics microgravity effects such as headward shifts of bodily fluids and axial body unloading [12]. HDBR can induce gait and balance impairment [13] and can result in structural brain changes [14, 15], changes in brain functional connectivity [16] and brain activation changes during performance of cognitive and motor tasks [17–19]. For example, following HDBR, gray matter (GM) volume increases and extracellular free water (FW) decreases in brain regions that control the lower limbs such as the para-cingulate gyrus. Larger increases in GM volume and larger decreases in FW [11] in these regions are associated with smaller decrements or even improvements in balance performance from pre to post HDBR, suggesting that such brain changes reflect compensatory processes. With HDBR there are also increases in functional connectivity (i.e., correlation of brain activation in distinct brain regions during rest) of vestibular, motor, and somatosensory brain networks, and decreases in connectivity of visual and somatosensory networks [16]. Larger connectivity increases between motor and somatosensory regions correlated with smaller decreases in balance performance with HDBR. Furthermore, HDBR leads to activation changes in the parietal operculum cortex in response to vestibular inputs. Larger increases in frontoparietal activation with HDBR correlated with greater HDBR-induced mobility declines [20]. Together, these findings could reflect neuroplastic mechanisms in response to the altered sensory inputs of the HDBR environment, some of which may facilitate adaptation. Furthermore, it should be noted that the above-described associations between brain changes and motor behaviour only make up for a smaller part of the widespread brain changes that are observed with HDBR. Thus, it is possible that at least some of the observed brain changes with HDBR are maladaptive. Therefore, CNS and motor dysfunction occurring in microgravity could jeopardize space mission success, and could also potentially interact with aging of brain structure and function in crewmembers. These changes are particularly concerning as spaceflight missions extend in duration and exploration targets are pushed beyond low Earth orbit where astronauts will be expected to ambulate autonomously in unfamiliar terrain.

These findings create a strong rationale to identify effective countermeasures for maladaptive brain changes (e.g., neurodegeneration) occurring with spaceflight and measures that promote compensatory brain changes occurring with spaceflight that could result in quicker readaptation to Earth’s gravity or other gravitational environments (i.e., Moon or Mars). Exercise during HDBR can mitigate effects on physical fitness [21], but it is not known what the effects are of exercise during HDBR on the brain. It has long been established that aerobic exercise in general can increase GM volume, potentially through dendritic branching, angiogenesis, synaptogenesis and gliogenesis [22]. Exercise further improves cognitive function through promotion of brain derived neurotrophic factor (BDNF), which plays a role in energy metabolism [23]. Such effects have been observed in both young and older adults [24, 25]. Moreover, exercise has a preventive effect on age-related neurodegeneration, an attenuating effect on neurological disease progression (e.g. traumatic brain injury and dementia) [26, 27], and results in improvements in cognitive functioning in subjects with mild cognitive impairment [28]. It is thus possible that exercise training could also mitigate the adverse effects of HDBR on brain structure and function. In support of this hypothesis, we and others previously showed that aerobic and resistance exercise partially mitigate the adverse effects of HDBR on gait and balance performance [13, 29, 30].

For the current study we analyzed effects of aerobic and resistance exercise on brain functional and structural changes from pre to post HDBR. Furthermore, we examined whether changes in physical fitness correlate with brain changes. The brain outcome measures were selected if they showed changes with HDBR in any of our previous studies, and include: 1) GM volume (T1 MRI data); 2) brain FW distribution (Diffusion Weighted Imaging; [11]); and 3) brain functional connectivity of sensorimotor regions (resting state fMRI; [16]). We selected the following physical fitness measures from the larger exercise study: 1) cardiorespiratory fitness (VO_2_ peak), 2) fat-free body mass, and 3) muscle structure and function. These measures have each been linked to brain structure and function in other studies [31, 32] and also exhibited significant changes from pre to post HDBR in non-exercise subjects. We hypothesized that a) exercise would mitigate a substantial part of the effects of HDBR on functional and structural brain changes and that b) changes in physical fitness would correlate with changes in brain function and structure of sensorimotor brain regions such as the primary motor cortex, the somatosensory cortex, the supplementary motor area, and the cerebellum.

## Method and materials

The current study is part of a larger prospective longitudinal HDBR framework study [33] for which subjects were randomized to a HDBR control group or a HDBR regular aerobic and resistance exercise group (see under ‘Bed rest exercise intervention’). Add-on studies to the HDBR framework study include a HDBR Flywheel group and MRI assessment of the brain. Here, we combine data from two study protocols that are embedded in the larger HDBR framework study, i.e., a study investigating physical activity as a countermeasure for musculoskeletal declines with HDBR [33] and a study investigating brain changes with HDBR [34].

### Participants

**Fig 1:**
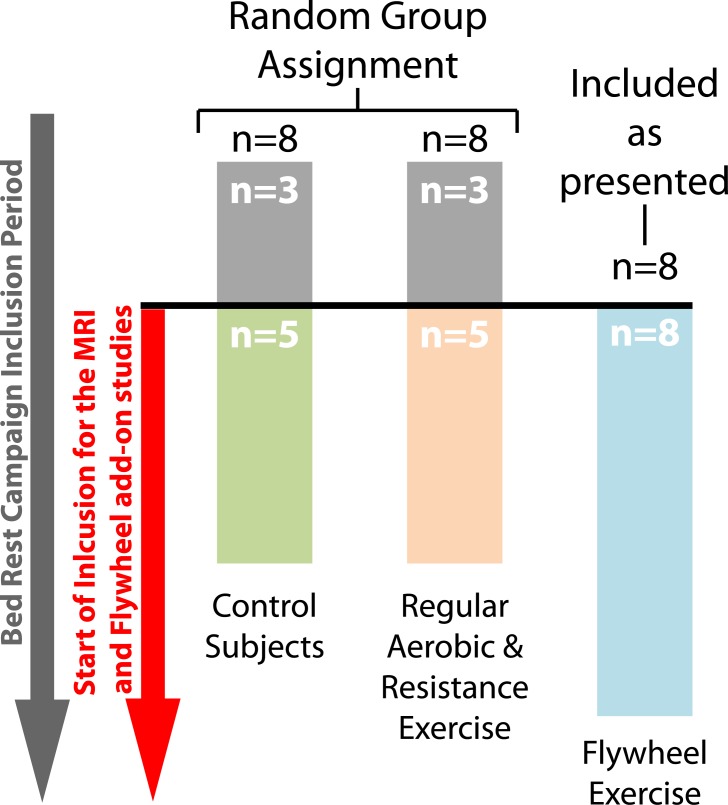
Subject inclusion flowchart. In total, 18 subjects participated in this study. These subjects were divided over 3 groups. Ten subjects were participants of the parent HDBR study and had been randomly assigned to either a HDBR control group or a HDBR regular aerobic and resistance exercise group. The remaining 8 subjects were sampled from an add-on study to the parent HDBR study. These 8 subjects completed flywheel exercise during the 70 days of HDBR and were included in the order they signed consent.

Inclusion of participants in the larger parent HDBR study started before the HDBR flywheel and HDBR neuroimaging study (see Fig 1). Here, we included only subjects that were enrolled in the parent HDBR study (i.e., HDBR control subjects and HDBR aerobic and resistance exercise subjects) and the HDBR flywheel add-on study who also participated in the HDBR neuroimaging add-on study. In total, 18 of the 24 participants completed both protocols. Six subjects (3 control subjects and 3 regular exercise subjects (see ‘Bed rest exercise intervention’) did not participate in the HDBR brain MRI study. The main outcomes from the HDBR neuroimaging study [11, 16, 19] and HDBR exercise study [35] have been reported separately.

The mean age of the 18 male subjects was 31.1 ± 4.7 years at time of admission (range: 25.7–39.8 years). Potential subjects were recruited via advertisement to the general public (i.e., nationally) and were prescreened by via an online form, or over the telephone by nurses of the NASA Test Subject Screening Facility. Inclusion criteria were 1) age between 24–55 years, 2) body mass index between 18.5 and 30.0 kg/m^2^, non-smoker, 3) no prescription medicine, 4) and absence of medical contraindications for participation in the study (e.g., contraindication for MRI). Subjects who passed the pre-screening underwent on-site screening that included an Air Force Class III equivalent physical examination, psychological examination, drug screening, and a criminal background check.

This study was conducted in compliance with the Declaration of Helsinki and approved by the following institutional review boards: 1) the University of Michigan; 2) the University of Texas Medical Branch (UTMB); and 3) NASA Johnson Space Center. All subjects provided written informed consent and received monetary compensation for their participation.

### Bed rest intervention

All subjects completed 70 days of 6°-HDBR at the bed rest facility located at the University of Texas Medical Branch (Galveston, TX). Subjects remained in the head down tilt position, except for 30 minutes during each meal (3 meals/day), when they were allowed to prop up their head. Subjects were admitted for baseline measures ~3 weeks before the start of HDBR and remained at the HDBR facility for another ~2 weeks after HDBR for follow-up assessments.

### Bed rest exercise intervention

Subjects were randomly assigned to a no-exercise control group (n = 5) or a traditional exercise group (n = 5). We also included subjects from an add-on study looking at the effects of Flywheel exercise on HDBR (n = 8). None of the participants dropped out of the study. Linear regression analysis did not show significant differences pre-HDBR between groups regarding age, height, weight or BMI (for all analyses: smallest *p* = .082; largest **η**^2^ = .19). Both exercise groups started familiarization with the exercise protocol 20 days before the start of HDBR. Intensity of training was gradually increased until the start of HDBR when the full exercise program began.

The exercise prescriptions have been described previously [36]. Both groups performed the same exercise prescription with the same intensity. The goal of comparing the two exercise groups was to evaluate the efficacy of a small exercise device that combines aerobic and resistive exercise for use on future exploration spaceflight vehicles (flywheel) compared to the suite of exercise devices available on the International Space Station (ISS) today (traditional) [37]. Therefore, one exercise group used traditional equipment similar to that found on the ISS and the other used a single compact flywheel rowing and resistance exercise device. The exercise prescription consisted of aerobic exercise six days per week and resistance exercise 3 days per week. For the traditional exercise group, aerobic exercise sessions consisted of alternating days of continuous cycle exercise for 30 min at 75% of VO_2_peak (3 days per week) with interval treadmill sessions of 30 s, 2 min, or 4 min intervals (3 days per week) at nearly maximal intensity. For the flywheel group, the aerobic exercise was completed using a compact flywheel rowing device for 30 min at 75% VO_2_max (3 days/week interval + 3 days/week continuous sessions). Resistance exercise was performed by both exercise groups every other day and consisted of 3 sets each of four supine lifts (squat, leg press, unilateral leg curl, and heel raise). After HDBR, all subjects began a rehabilitation program consisting of 1h daily aerobic and resistance exercise. Because the exercise intensity of both programs are highly similar, in order to increase power, we combined the two exercise groups into one exercise group for our statistical analysis (see under statistical analysis).

### Assessment, processing, and analysis of physical fitness measures

**Fig 2:**
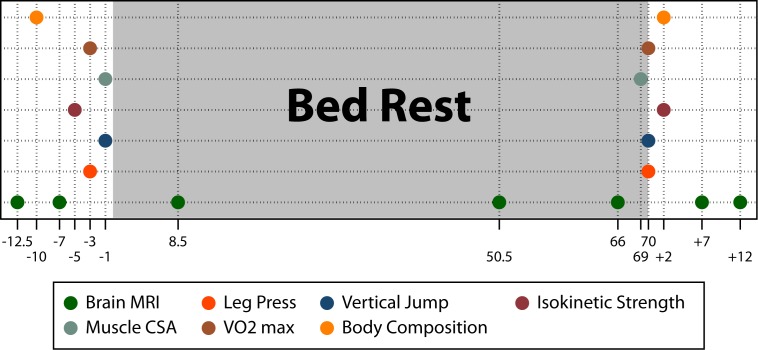
Time line of data collection of brain MRI and physical fitness measures. The x-axis indicates the number of days relative to the HDBR intervention (e.g., -12.5 = 12.5 days pre-HDBR; 8.5 = days in HDBR; and +2 = 2 days post-HDBR). Brain MRI = structural T1-weighted MRI, diffusion weighted imaging, and resting state functional connectivity MRI; Leg Press = lower body strength (isometric, isokinetic, total work); Vertical Jump = vertical jump (height and Watt) Isokinetic Strength = isokinetic knee and ankle extension and flexion strength; Muscle CSA = muscle cross-sectional area (soleus, quadriceps, hamstrings); VO2 max = rate of oxygen consumption during peak performance; Body Composition = fat mass and fat free mass.

For an overview of data collection time points, see Fig 2.

#### Lower body muscle performance

To measure lower body isometric strength, subjects performed 3 maximal efforts for 5 s each with 30 s of rest between each effort. To assess upper and lower body dynamic power and work capacity, subjects performed 21 consecutive ballistic, concentric-only bilateral leg press actions with the load fixed at 40% of the measured maximal isometric force, which has previously been shown to elicit maximal power output.

#### Vertical jump

Subjects performed three maximum effort jumps with 60–90 s of rest between each jump. All jump trials were performed on a force plate and data were acquired using a custom software program using a sampling rate of 1000 Hz (LabVIEW, National Instruments). Acceleration profile, jump height, peak acceleration, peak velocity, and peak power were calculated. Acceleration profile was calculated by dividing the vertical ground reaction force by body mass. To calculate jump height a double integration of the acceleration profile was computed.

#### Isokinetic leg strength

Knee and ankle extension/flexion muscle strength were measured with an isokinetic dynamometer (Biodex System 4, Biodex Medical Systems, Shirley, NY) following the same protocol used by our group previously [36]. Knee and ankle extensor/flexor peak torque production was assessed during maximal repetitions performed at 60°/s and 30°/s, respectively.

#### Muscle MRI

Cross sectional area (CSA) of the lower leg muscles was obtained from MRI scans pre and post-HDBR. Images were acquired from the level of the ankle mortise to the iliac crest. The methods and reliability of this technique have been previously reported by our laboratory [38]. Muscle CSA was manually traced using Image-J (National Institutes of Health, Bethesda, MD, USA, version 1.42).

#### Maximal aerobic capacity and ventilatory threshold

Aerobic capacity (VO_2peak_) was assessed during upright peak cycle ergometry before and after the bed rest period as previously described [36].

#### Bone and body composition assessments

Body mass was obtained daily (Cromwell et al., under review as a companion paper). Dual-energy x-ray absorptiometry scans (DXA) were obtained pre-, in- and post-HDBR. Scans included whole body analysis of bone mineral density (BMD).

Changes in physical fitness measures were analyzed using linear regression analysis with percent change score from pre-HDBR to post-HDBR as outcome measure and group (3 levels) as a covariate of no interest. Differences between HDBR exercise subjects and HDBR control subjects were analyzed using linear regression analysis with group (exercise vs. control) as predictor and type of exercise as covariate of no interest.

### Brain image acquisition and processing

For the current study we analyzed whole brain maps of 1) GM volume derived from T1-weighted MRI; 2) FW derived from diffusion weighted MRI; and 3) functional connectivity derived from resting state fMRI using selected sensorimotor seed regions. These MRI outcome measures were selected because we previously found them to be sensitive to HDBR in this sample [11, 16, 19].

All MRI data were collected at 7 time points pre, during, and post-HDBR using a 3-Tesla Siemens Magnetom Skyra MRI scanner and a 32 channel head coil. A 3D T1 sagittal MP-RAGE sequence was used to collect high-resolution T1-weighted images (in plane resolution: 0.94×0.94 mm; slice thickness 0.90 mm). The longitudinal pipeline of the Voxel Based Morphometry 8 add on for the Statistical Parametric Mapping 8 toolbox running under MATLAB R2014a was used to obtain probabilistic GM maps from the T1 data and to normalize these images to Montreal Neurological Institute (MNI) standard space. All normalized GM maps were smoothed with an 8 mm full-width at half-maximum Gaussian kernel to increase signal to noise ratio.

2) 62 Diffusion weighted images (2 b = 0 s/mm^2^ and 60 b = 1000 s/mm^2^ images in 30 non-collinear directions) were collected using a diffusion-weighted 2D echo-planar imaging sequence (in plane resolution: 1.875×1.875 mm; axial slice thickness 2.00 mm; scan duration ~10 minutes). A Rician filter was applied under MATLAB to these images to remove random noise. Subsequently, eddy current and b-vector adjustment were applied in FMRIB Software Library (FSL 5.0.8). We then manually checked and removed all volumes with artifacts and excessive head motion. Next, we applied a free water imaging model using in-house developed MATLAB code [39]. The algorithm separately models water molecules that are free to diffuse and water molecules that are hindered or restricted by cellular barriers. This results in maps of FW describing the fractional volume of freely diffusing molecules in each image voxel. To register these FW images to MNI standard space we used a longitudinal pipeline [11] implemented in Advanced Normalization Tools (ANTs). The images in MNI space were smoothed to increase signal to noise ratio with a Gaussian kernel that had a standard deviation of 3.4mm, equivalent to ~8 mm full-width at half-maximum.

3) For resting state functional connectivity MRI we collected 164 volumes using single-shot gradient-echo echo planar imaging (in plane resolution: 2.55×2.55 mm; axial slice thickness 5.00 mm; scan duration ~10 minutes). The raw fMRI data were corrected for slice timing and realigned for head motion using SPM8. The T1-weighted images were then co-registered to the subject’s mean realigned fMRI image and subsequently normalized to MNI space using ANTs. The obtained warp parameters were used to bring the fMRI data into MNI common space. Next, the normalized data were smoothed using a Gaussian kernel with a standard deviation of 4 mm (~9.4 mm FWHM). Functional connectivity maps were obtained using the CONN toolbox with default settings. For the current study we selected the following seed regions of interest (ROI) that yielded significant network connectivity changes over the course of HDBR based on one of our previously published experiments of the entire group of HDBR subjects [16]: the a) left primary motor cortex (MNI coordinate -38,-26,50); b) right operculum parietale 2 (MNI coordinate 42,-24,18. Operculum parietale 2 has been shown to play a key role in vestibular processing [40, 41]); c) right superior parietal gyrus (posterior parietal cortex; MNI coordinate 28,-72,40); d) right cerebellum lobule VIIIb (MNI coordinate 20,-57,-53); and e) right cerebellum lobule V (superior posterior fissure; MNI coordinate 24,-81,-36). ROIs were defined as 4mm spheres centered on coordinates within sensorimotor brain regions. Connectivity maps were constructed by computing the bivariate correlation between the average time series of each ROI and all other voxels in the brain. In addition, we included whole brain intrinsic connectivity maps, which represent for each voxel in the brain the average connectivity strength (r^2^) with all other voxels in the brain.

For one subject at one time point MRI data were lost during data transfer. For two HDBR control subjects, diffusion weighted MRI data was not collected pre-HDBR. These subjects were therefore excluded from statistical analyses.

### Voxel-wise group analysis of brain changes with HDBR

To assess changes between groups over time from a) pre-HDBR to the end of HDBR and b) from the last assessment time point in HDBR to the last post-HDBR time point we calculated per subject and per outcome measure a regression slope map. These maps represent the daily amount of linear change. For the GM and FW measures we subsequently divided these slope maps by the individual intercept maps to obtain proportional change maps. Functional connectivity slope maps were not divided by their intercepts because this would hamper the interpretation, due to the fact that intercept connectivity maps can contain both negative and positive values. Dividing them can change the direction of the effect. To take into account any group differences at baseline for the functional connectivity maps we analyzed between group differences at baseline (i.e, ~8 days pre-HDBR) for all functional connectivity maps. Except for stronger functional connectivity between the operculum parietale 2 ROI and the inferior occipital gyrus in HDBR control subjects than in HDBR exercise subjects there were no significant differences (all *p*>0.05 family-wise error corrected). Because of the absence of significant differences between groups at baseline, we compared the slope images directly.

We used non-parametric permutation based models implemented in FSL’s ‘randomise’ with 15,000 random permutations, threshold-free cluster enhancement and variance smoothing (8mm full width at half maximum) for inference. To test for the effects of exercise during HDBR we modeled each of the three HDBR groups separately in our design matrix and compared the HDBR control group to the combined HDBR exercise groups in our contrast matrix. Alpha levels were set at *p*<0.05 adjusted for multiple comparisons through family-wise error correction. Family-wise error correction corrects for the number of tests within each voxel-wise analysis, but not for the number of voxel-wise analyses.

We tested for group differences between the HDBR regular aerobic and resistance exercise group and the HDBR flywheel exercise group to validate the pooling of these exercise groups in subsequent analyses. For the analyses we used the above described voxel-wise permutation-based analyses with the same settings. There were two small regions in which we observed differences between the two exercise groups during the period from pre-HDBR to the end of HDBR: 1) There was a connectivity increase between right cerebellum lobule V and the left intracalcarine cortex in regular exercise subjects but a connectivity decrease between these regions in flywheel subjects (cluster size: 43 voxels (8mm^3^/voxel): MNI coordinate: -16, -78, 10); 2) There was a connectivity decrease between the right superior parietal gyrus and the right precuneus in regular exercise subjects and a connectivity increase between these regions in flywheel subjects (cluster size: 11 voxels, MNI coordinate: 6, -56, 50). No other significant differences between HDBR exercise groups in any other brain regions for any of the outcome measures at any of the time periods (pre-HDBR to the end of HDBR and end of HDBR to post-HDBR) were observed. We therefore concluded that it was valid to pool the exercise groups for subsequent group comparisons with the HDBR control group.

### Voxel-wise correlational analysis between brain changes and physical fitness changes

To reduce the number of tests we selected one outcome measure per set of physical fitness measures (i.e., leg press, vertical jump, isokinetic force, muscle CSA, VO_2_ max, body composition). We selected those outcome measures that showed the largest percent change from pre-HDBR to within 2 days post-HDBR and which were collected for all subjects. This resulted in the following: 1) Isokinetic leg press total work over 20 reps (lbs); 2) Vertical jump distance (cm); 3) Knee extension strength (nM); 4) Soleus muscle CSA (cm^2^); 5) maximum rate of oxygen consumption during peak cycle (VO_2_ max, l/min); 6) Fat mass as percentage of total body weight; and 7) Oxygen intake during rest (l/min). Changes with HDBR in these outcome measures have been reported previously [36]. An overview is presented in Table 1.

**Table 1:** Physical fitness changes with bed rest.

Outcome	Total Sample (n = 18) ^1^					HDBR Control Subjects (n = 5) ^2^		HDBR Exercise Subjects (n = 13) ^2^		Control vs. Exercise ^2^	
	Mean Pre HDBR	Mean Post HDBR	% Change	se % Change	*p* % Change	% Change	*p* % Change	% Change	*p* % Change	*p* Group Difference	*Cohen’s D*
Leg press	9380.1	8554.1	-8.4	2.56	0.004	-20.0	<0.001	-1.2	0.77	0.003	1.78
Jump	0.6	0.5	-8.0	2.27	0.003	-20.1	<0.001	-5.4	0.012	0.001	1.45
Knee	211.5	185.1	-12.1	2.53	<0.001	-20.3	<0.001	-3.0	0.28	0.008	1.56
Soleus	26.1	22.6	-13.4	1.84	<0.001	-24.8	<0.001	-8.1	<0.001	<0.001	2.86
VO_2_ max	3.1	2.8	-8.7	2.46	0.003	-17.1	0.001	-6.6	0.056	0.10	0.90
Fat mass	20.7	21.7	3.74	1.87	<0.001	11.1	<0.001	6.7	0.022	0.20	0.44

HDBR = head down bed rest; se = standard error; Leg press = isokinetic leg press total work over 21 reps (lbs); Jump = vertical jump (meter) divided by body weight (Kg); Knee = knee extension strength (nM); Soleus = Soleus muscle cross sectional area (cm^2^); VO_2_ max = maximum rate of oxygen consumption during peak cycle (l/min); Fat mass = Fat mass percentage of total body weight;

^1^ = analyzed using t-tests for the combined sample;

^2^ = analyzed using regression analysis with group (exercise vs. control) as predictor and type of exercise as covariate of no interest

We analyzed the association between changes in physical fitness measures and changes in the predefined nine MRI outcome measures. To this end we used non-parametric permutation based models implemented in FSL’s randomise with 15,000 random permutations, threshold-free cluster enhancement and variance smoothing (8mm) for inference. Associations between changes in fitness measures and brain measures were tested by testing the association between the percent change maps (for GM/FW) and regression slope maps (for functional connectivity) with difference scores (post-HDBR—pre-HDBR) of the physical fitness measures. Because we were interested in the association between these associations regardless of whether subjects exercised during HDBR, we used linear regression analysis with HDBR group (3 levels) as covariate of no interest to regress out the difference in fitness change between groups. Alpha levels were set at 0.05 adjusted for multiple comparisons through family-wise error correction. Family-wise error correction corrects for the number of tests within each voxel-wise analysis, but not for the number of voxel-wise analyses.

## Results

### Physical fitness changes with HDBR

Physical fitness declined significantly from pre to post-HDBR on all indices in the total sample (see Table 1). The percent fitness decline adjusted for exercise group was approximately 20% and ranged from 11.1% (increase in fat mass) to 24.8% (loss of soleus muscle CSA). Exercise significantly reduced the effects of HDBR on all physical fitness indices except for VO_2_ max and fat mass (all p’s < .01 except for VO_2_ max where p = 0.10 and fat mass where p = 0.20; see Table 1 for exact p values, percent change in fitness measures, and effect sizes). The effect sizes (Cohen’s D) for fitness change during HDBR in controls versus exercisers ranged from 1.45 to 2.86 and can all be considered very large. The exercise effects in the full exercise study sample are further elaborated in another paper [33]. The results largely parallel what is reported here for the reduced data set.

### Voxel-wise group analysis of brain changes with HDBR

No significant differences were observed in pre to post-HDBR changes between the combined exercise group and the control group in focal gray matter volume, FW content, or functional connectivity.

**Fig 3:**
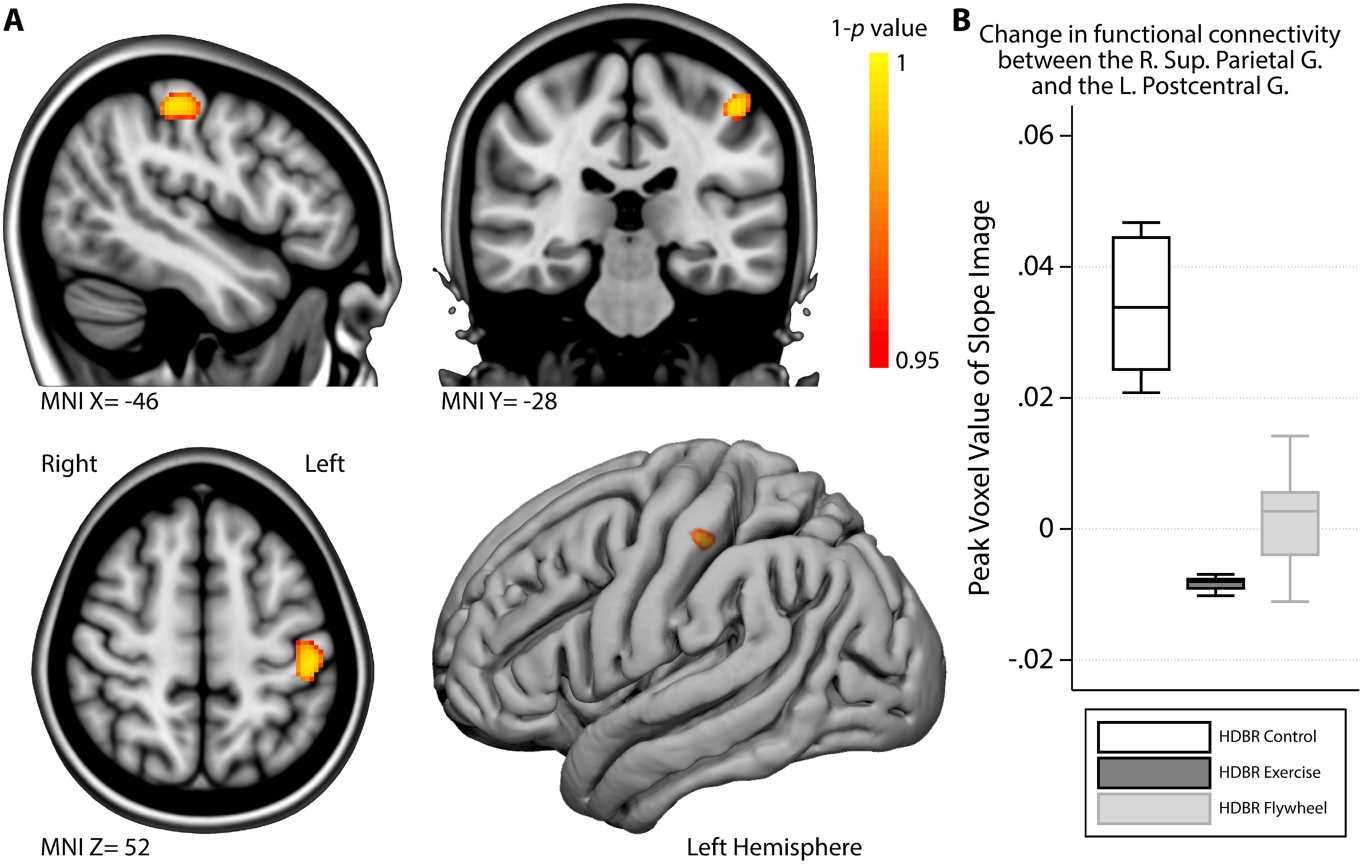
Exercise modulates functional connectivity strength between the right superior parietal gyrus and the left postcentral gyrus post-HDBR. A) Regions in which HDBR control subjects showed a significantly larger increase in functional connectivity between the Right Superior Parietal Gyrus and the Left Postcentral Gyrus than the HDBR exercise subjects during the post-HDBR phase. B) Boxplot of the functional connectivity strength measure (daily change in correlation) of the most significant ‘peak’ voxel in the area depicted in A, stratified by group. MNI = Montreal Neurological Institute coordinate; R. = Right; L. = Left; G. = Gyrus.

The change in functional connectivity from the end of HDBR to the last assessment post-HDBR between the right superior parietal gyrus seed region and the left postcentral gyrus was significantly larger in HDBR control subjects than in HDBR exercisers (see Fig 3A; please note that for the analyses the exercise groups were combined). We extracted information from the voxel that differed most significantly between the combined exercise groups and the control group from each of the three groups separately to help with the interpretation; Whereas the exercise group showed little to no change in connectivity during recovery, control subjects showed an increase in connectivity (see Fig 3B). Because we did not observe significant between group changes for functional connectivity with the superior parietal gyrus seed region during HDBR, we conducted the following post-hoc steps to help interpret the current finding: 1) we located the peak (most significant) voxel of the analysis; 2) we obtained the functional connectivity metric at the peak voxel from all subjects’ pre-HDBR maps and compared these values between the groups using linear regression analysis; 3) we did the same for the subjects’ slope maps that indicate the magnitude of change from pre-HDBR to the end of HDBR. These analyses revealed that pre-HDBR, there were no group differences in functional connectivity between the right superior parietal gyrus seed region and the left postcentral gyrus, but control subjects showed a significant decrease in functional connectivity between these regions from pre-HDBR to the end of HDBR (z-score_(margin control subjects)_ = -0.90, t = -2.34, *p* = .034) that was not present in the exercise subjects (z-score_(margin exercise subjects)_ = .34, t = 1.47, *p* = .16). No other significant between group differences were observed in the rate at which focal gray matter volume, FW content, or functional connectivity changed from the end of HDBR to the second (i.e., last) assessment time point post-HDBR.

### Voxel-wise correlation between brain changes and physical fitness changes

**Fig 4:**
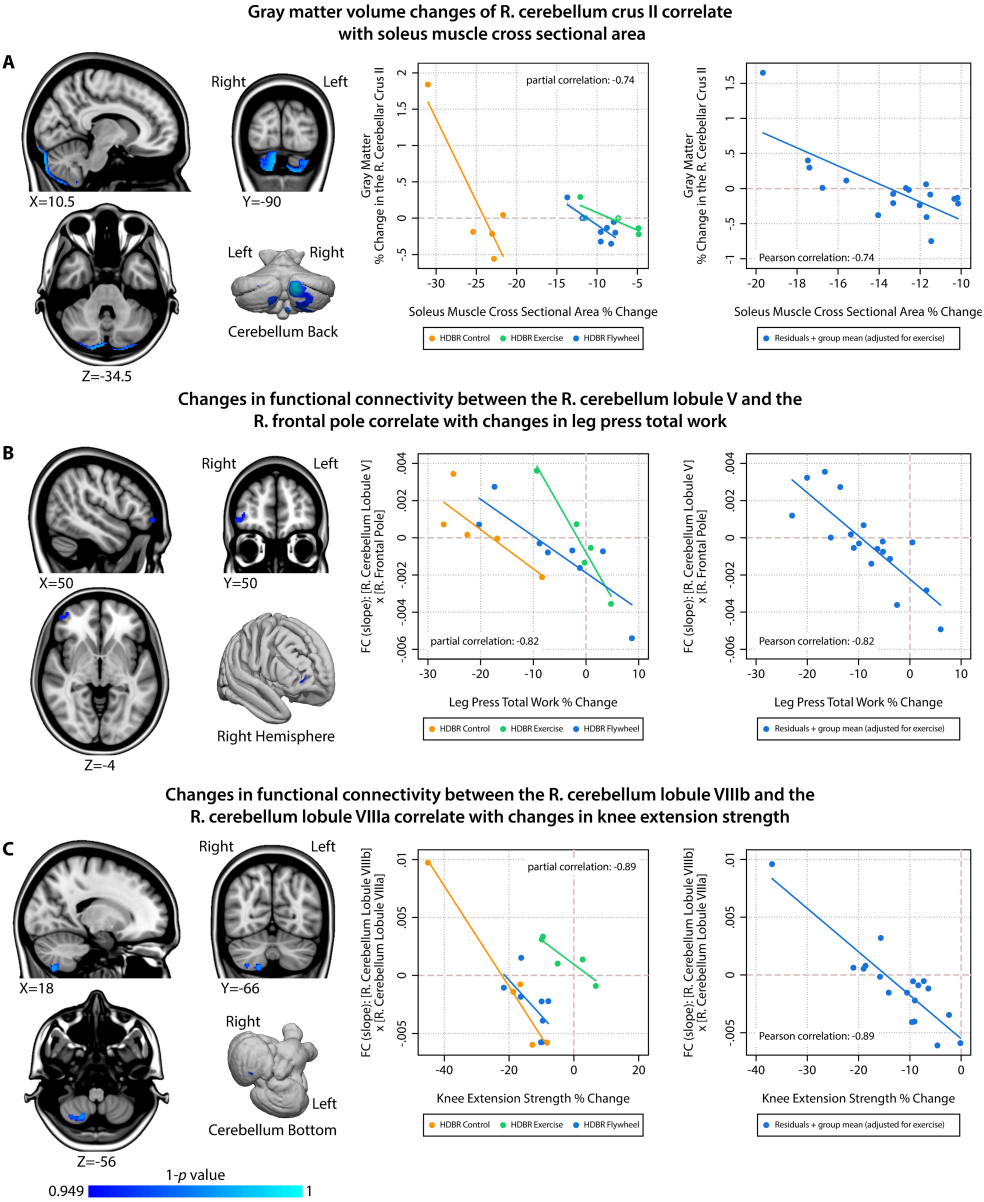
Associations between HDBR-induced changes in brain outcome measures and changes in physical fitness (part 1). Left column = overview of locations showing significant associations between changes in physical fitness and changes in brain measures; Middle column = scatterplot with fit lines showing correlations at the peak voxel stratified by HDBR group; Right column = scatterplot with fit line showing correlations at the peak voxel adjusted for exercise; R. = Right; FC = Functional Connectivity.

**Fig 5:**
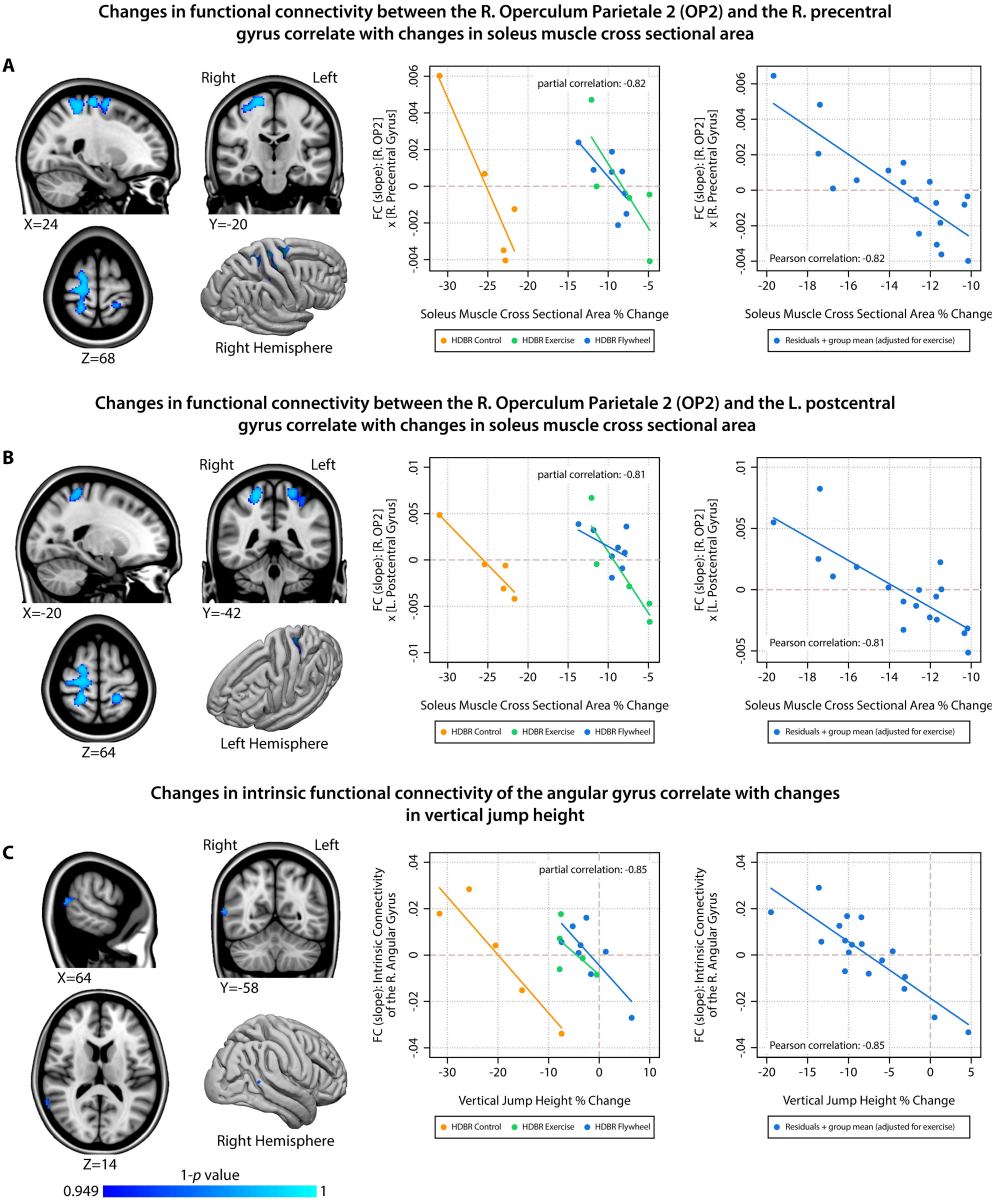
Associations between HDBR-induced changes in brain outcome measures and changes in physical fitness (part 2). Left column = overview of locations showing significant associations between changes in physical fitness and changes in brain measures; Middle column = scatterplot with fit lines showing correlations at the peak voxel stratified by HDBR group; Right column = scatterplot with fit line showing correlations at the peak voxel adjusted for exercise; R. = Right; FC = Functional Connectivity; L. = Left.

Because we observed group-by-time interactions for the physical fitness measures, we adjusted for exercise group when examining associations between fitness changes and brain changes. Therefore, significant correlations between fitness changes and brain changes can be interpreted as overall effects (regardless of group). An overview of the significant correlations between brain and physical fitness changes with HDBR is presented in Table 2, Figs 4 and 5. All associations between changes in physical fitness and brain measures comprise a negative slope that crosses zero (indicating that there are always subjects who show increases as well as subjects who show decreases in brain measures). Gray matter volume changes in cerebellar lobule Crus II correlated with changes in soleus muscle CSA (Fig 4A). We observed negative correlations between: functional connectivity between right cerebellum lobule V and the right frontal pole with changes in leg press total work (Fig 4B); functional connectivity between the right cerebellar lobule VIIIb and the right cerebellar lobule VIIIa with changes in knee extension strength (Fig 4C); functional connectivity between the right operculum parietale 2 and the right precentral gyrus (Fig 5A) as well as the left postcentral gyrus (Fig 5B) with changes in soleus muscle CSA; and intrinsic functional connectivity of the angular gyrus with changes in vertical jump height. All of these significant correlations were negative, indicating that larger decreases in physical fitness were associated with smaller decreases or even increases in gray matter volume or functional connectivity. For each significant finding, we provide partial correlations (i.e., total group correlations adjusted for exercise group) for the voxel that is most significantly associated with changes in physical fitness as an indication of effect size (see the scatterplots in Figs 4 and 5). None of the other combinations of brain changes and physical fitness changes were significant.

**Table 2:** Labels and peak coordinates of significant clusters from physical fitness correlational analysis.

MRI Measure	Physical fitness measure	Cluster Size	MNI Coordinate of Local Maxima				
			X	Y	Z	Max T-Score	Anatomical Label
Gray Matter Volume	Soleus Muscle CSA	3032	10.5	-90	-34.5	6.4	R. C. Crus II
			18	-93	-36	6.3	R. C. Crus II
			9	-88.5	-42	6.1	R. C. Crus II
			-7.5	-85.5	-48	6.0	L. C. Crus II
			16.5	-75	-58.5	5.7	R. C. Lobule VIIb
			27	-85.5	-49.5	5.2	R. C. Crus II
FC: R. C. Lobule V	Leg Press Total Work	74	50	50	-4	4.5	R. Frontal Pole
			40	54	4	4.3	R. Frontal Pole
FC: R.C. Lobule VIIIb	Knee Extension Strength	114	18	-66	-56	5.8	R. C. Lobule VIIIa
			32	-66	-54	4.9	R. C. Lobule VIIb
FC: R. Operculum Parietale 2	Soleus Muscle Volume	1432	24	-20	68	5.0	R. Precentral Gyrus
			22	-40	64	5.2	R. Postcentral Gyrus
			20	-6	68	4.6	R. Superior Frontal Gyrus
			22	-6	56	3.5	R. Superior Frontal Gyrus
			44	-28	54	3.5	R. Postcentral Gyrus
			40	-28	44	3.6	R. Postcentral Gyrus
		333	-20	-42	64	5.2	L. Postcentral Gyrus
			-32	-40	54	4.3	L. Superior Parietal Lobule
FC: Whole Brain Intrinsic Connectivity	Vertical Jump Height	52	64	-58	14	5.8	R. Angular Gyrus

MNI = Montreal neurological institute; GM = gray matter volume; FC [] = functional connectivity [seed region]; C. = cerebellum; L. = left; R. = right cluster size is in voxels; CSA = cross sectional area; Labels are derived from the Harvard-Oxford Cortical Atlas [42] and the SUIT cerebellum atlas [43]; Only local maxima with at least 1cm distance are listed

## Discussion

We evaluated exercise as a potential countermeasure for the effects induced by a spaceflight analog on the brain. Considering the robust effects of spaceflight on brain structure, function, and motor behavior that we and others have observed in astronauts and in individuals in a microgravity analog environment, there is need for targeted countermeasures. We investigated whether aerobic and resistance exercise mitigates the effects of HDBR on brain structure and function and if HDBR-induced changes in a variety of physical fitness measures correlate with HDBR-induced changes in brain structure and function. In contrast to our first hypothesis, we found limited evidence for the former. However, in line with our second hypothesis, we observed several significant associations between deterioration of physical fitness such as muscle strength and muscle volume and brain structural and functional changes with HDBR, for example in the cerebellum and the pre- and post-central gyrus. HDBR is a microgravity analog, and as such, these results provide new pointers for studying the role of central nervous system changes in motor behavioral deficits in astronauts upon return to Earth.

Within our sample, exercise significantly mitigated changes in physical fitness, but it did not significantly moderate brain structural or functional changes from pre-HDBR to the end of HDBR, Perhaps, although our sample size was sufficient to pick up the larger effects of exercise on physical fitness, it was insufficient to detect effects on brain changes during HDBR. However, during the post-HDBR recovery phase, control subjects showed a significant increase in functional connectivity between the right superior parietal gyrus and the left postcentral gyrus, whereas the HDBR exercise subjects showed almost no changes in functional connectivity between these regions. Posthoc analysis indicated that the connectivity increase in HDBR control subjects post-HDBR reflects a recovery following a significant decrease in connectivity between the two regions during HDBR in these subjects. Such a change during HDBR was not observed in exercise subjects. This further indicates that a with a larger sample size we may have been able to pick up effects of exercise during HDBR and that some effects may have been masked out due to the statistical corrections for the whole brain analyses. The superior parietal gyrus is involved in mental orientation in space, time, and person, and the postcentral gyrus processes somatosensory information. These results could therefore indicate that the recovery in functional connectivity in the HDBR control subjects reflects re-adaptation to the upright environment. The fact that no significant voxel-wise between-group differences in functional connectivity of these brain regions occurred during HDBR could be due to our small sample size, or larger variation in change within subjects from pre- to the end of HDBR compared to during the post-HDBR recovery period. Considering that we did not observe any other effects of exercise on brain structural and functional outcome measures that we have previously shown to be affected by HDBR [11, 16, 19], we conclude that aerobic and resistance/flywheel exercise is not a very strong countermeasure for the effects of HDBR on brain structure and function. Nevertheless, considering that our posthoc analysis showed a positive effect of exercise on brain functional connectivity, studies with larger sample sizes are necessary to determine if such associations are substantial and if they are observed in other regions and with other MRI modalities as well.

Correlational analyses showed that, the magnitude of physical fitness changes was associated with brain structural and functional changes across individuals in a fashion that suggests that supine exercise may reduce demand for compensatory HDBR-induced brain changes. Large fitness decreases were associated with connectivity and volumetric increases, potentially reflecting some compensatory process. In fact, those who show no fitness loss, or small fitness gains exhibit modest brain changes, or even decreases in connectivity and volume. Larger studies are warranted to investigate this in the future.

A larger decrease in soleus muscle size was associated with a smaller decrease or even an increase in gray matter volume in cerebellum crus II and lobule VIIb. These regions have mainly been associated with cognitive functions [44]. However, gray matter decreases were also present in a substantial part of cerebellar lobule VIIIb, which is a sensorimotor region [45]. Potentially, muscle degeneration could affect the proprioceptive input that the cerebellum receives, especially since HDBR adversely impacts balance [13] and because the soleus muscle is important for posture control. Previous studies have shown that limb immobilization can result in gray matter decrease in brain regions controlling that limb [46]. The decreases in gray matter could reflect loss of dendrites and their synapses [47]. However, our results show that larger muscle loss is associated with smaller brain changes. Potentially, proprioceptive input is required for more adaptive change, but there is less change with more muscle mass loss.

Changes in physical fitness measures did not correlate with changes in brain extracellular water content. However, we did observe relationships between changes in physical fitness during HDBR and changes in functional connectivity. Subjects with larger decreases in leg press total work showed more connectivity increases between cerebellum lobule V and the frontal pole. Lobule V is among others activated during tactile stimulation of the foot [45] and involved in regulation of force amplitude. The frontal pole is involved in goal-directed behavior. It may therefore be possible that the increased connectivity between these regions represents compensation for the HDBR environment and reduced strength, which may in turn benefit leg press performance. In other words, by having different brain regions working more closely together on the same task, the brain tries to maintain the level of physical performance. This idea is in line with studies that showed that increases in functional connectivity between motor brain regions correlate with motor recovery after stroke [48]. Other associations that we observed also suggest neural compensation for deterioration of physical fitness with HDBR. For instance, the association between loss of knee extension strength and increases in connectivity of right cerebellar lobules VIIIb and VIIIa. Like lobule VIIIb, lobule VIIIa is involved in motor control [45]. Perhaps, the brain tries to improve motor control by coactivating these cerebellar motor areas as adaptation to the HDBR environment, which subsequently mitigates loss of knee extension strength.

The most widespread cerebral changes in our study were observed in relation to HDBR induced reductions in soleus muscle size. Not only did loss of soleus muscle size correlate with gray matter changes (see above), it also correlated with decreases in functional connectivity of the operculum parietale 2 with widespread cortical regions, including the left precentral gyrus and the bilateral postcentral gyrus. The operculum parietale 2 which is part of the vestibular cortex that processes vestibular and proprioceptive inputs and plays an important role in balance control [49]. The precentral gyrus is involved in execution of movement, while the postcentral gyrus processes somatosensory information. Together, these regions make up an integrated system that is crucial for sensorimotor control. It is plausible that HDBR induced changes in soleus muscle size affects the proprioceptive input to these brain regions. Lastly, we observed an association between decrements in vertical jump height and smaller decreases or even increases in intrinsic connectivity of the right angular gyrus. Intrinsic connectivity changes indicate that a region is overall more/less (depending on the direction) connected to all other regions in the brain. The angular gyrus plays a role in sensorimotor integration [50]. The observed association could be explained as a compensatory mechanism in which more loss of vertical jump could result in larger changes in the processing and integration of multisensory information in an attempt to maintain performance.

Previous analysis of cognitive data that was collected for this sample showed no association between HDBR and cognitive functioning or exercise during HDBR and cognitive functioning [13]. We have therefore not controlled for cognitive functioning in our analyses.

A limitation of our study is its small sample size which likely has affected the possibility to detect effects of exercise on brain structure and function during HDBR. The complexity of our prospective longitudinal study that includes a bed rest and an exercise intervention with physical fitness measures and MRI data collections at multiple time points makes it a time consuming and logistically challenging project. This limits the number of subjects that can be included within a reasonable timeframe. Future studies should therefore aim at pooling data or setting up multicenter studies to increase sample size. Also, the sample of HDBR subjects is ~10 years younger than the average age of astronauts. It could therefore be that the effects of exercise on brain function and structure that we report on here do not apply in the exact same way to the generally older astronaut population. However, studies on Earth have shown beneficial effects of aerobic exercise on brain health in aging populations and even in those with neurodegenerative diseases, indicating that exercise also promotes brain health in older individuals [51, 52].

All our voxel-wise analyses were corrected for multiple comparisons at the voxel level (i.e., to adjust for the multiple tests carried out within each brain) by controlling for the family wise error rate. The results from these analyses did not survive additional Bonferroni adjustment to account for the number of different tests that were conducted (i.e., the number of associations between brain and physical fitness outcome measures that were tested). Although these results should therefore be interpreted with caution, the overall pattern of correlations between HDBR induced changes in physical fitness and changes in functional connectivity of sensorimotor brain regions, and the fact that these correlations were all strong (all: r < -0.81), provides evidence for the idea that loss of physical fitness with HDBR is associated with brain changes with HDBR. The correlational approach does not answer any questions of causality. It could be that physical fitness changes affect brain functional and structural plasticity. This could explain the association between loss of muscle size and changes in brain outcome measures. Alternatively, changes in sensorimotor brain regions could affect the neural control of movement, resulting in for example suboptimal performance in a vertical jump task. It is however most likely that the changes in the brain and in physical fitness with HDBR interact with each other, because 70 days of HDBR is an intervention that has a significant impact on multiple physiological systems, (e.g., the cardiovascular, skeletal, and muscle [53], visual [54], and central nervous system [11, 16, 19]) that rely heavily on each other.

The associations between brain and physical fitness changes with HDBR provide a better understanding of the motor behavioral changes that astronauts present with upon return to Earth. Future studies could focus on whether physical fitness changes predict neural changes, and vice versa, to determine causality, and to help identifying those individuals that would be most affected by bed rest, hospital based deconditioning or spaceflight and who would benefit most from exercise therapy.
